# Supplementary material for: Optical emission near a high-impedance mirror
Source: Nat Commun. 2018 Aug 13;9:3224. doi: 10.1038/s41467-018-05505-w (PMC6089971; doi:10.1038/s41467-018-05505-w)
Supplement: Supplementary file 1 — Supplementary Information [file 41467_2018_5505_MOESM1_ESM.pdf]

Supplementary information materials

Optical emission near a high-impedance mirror

Majid Esfandyarpour, Alberto G. Curto, Pieter G. Kik, Nader Engheta, and Mark L. Brongersma

Table of Contents

Sections

Supplementary Note 1: Emission properties of a dipole near a smooth and a patterned surface ..2

Supplementary Note 2: SPP transmission and reflection across a finite number of grooves …....5

Supplementary Note 3: Electrical properties of HIM electrodes …………………..……………6

Supplementary Figures

Supplementary Fig. 1. The relevant decay channels for an electric dipole emitter near a metal surface

Supplementary Fig. 2. Optical simulation of the electric dipole emission near smooth and patterned metal surfaces.

Supplementary Fig. 3. Height dependence of the loss ratio for electric dipoles above a flat Ag film and a high impedance metasurface.

Supplementary Fig. 4. Impact of single and multiple grooves on the dissipation via SPPs and LWs

Supplementary Fig. 5. Far-field angular radiation pattern for an electric dipole above a smooth electric mirror (EM) and a high-impedance metasurface (HIM).

Supplementary Fig. 6 Optical simulation of surface plasmon transmission and reflection across finite number of subwavelength grooves.

Supplementary Fig. 7. Time-resolved photoluminescence measurement of R6G dye molecules above different metallic surfaces.

Supplementary Fig. 8. Band structure calculation for a periodic dimple array

Supplementary Note 1. Emission properties of a dipole near a smooth and a patterned surface

In this section, we compare the strength of the undesired coupling of an electric dipole emitter to surface plasmon polaritons (SPPs) and Lossy Waves (LWs) for cases where it is placed above a smooth silver (Ag) film or a patterned high-impedance metasurface. To argue the importance of both types of optical excitations in limiting the emitter's efficiency, we start with a quantitative analysis of the relevant decay channels for electric dipole emitters above a flat Ag mirror. Following the approach in reference 4, we can quantify the relative decay rates/efficiencies for the coupling into the two unwanted loss channels mentioned above as well as the desired free-space radiation channel. Supplementary Fig.1 plots these efficiencies as a function of the emitter spacing above the mirror when the emission wavelength is 560 nm and for dipole orientations that are either parallel or orthogonal to the metal surface. It highlights the importance of the non-radiative SPPs and LWs channels for spacings that are a small fraction of the emission wavelength (< 100 nm). The figure shows that for a dipole spacing around 10 nm the coupling rates to SPPs and LWs become comparable while at distances well in excess of 10 nm the SPP loss channel dominates over LWs. As note of caution, in the literature related to organic LEDs the losses to SPPs and LWs are typically not separated out and are both referred to as SPP loss.

This work discusses how high impedance metasurfaces (HIMs) can suppress both loss channels to improve the overall external quantum efficiency of the thin light-emitting device. A detailed look at the field distribution near emitters placed above smooth metal surfaces and HIMs can provide insights into the mechanisms that give rise to the quantum efficiency enhancements. Supplementary Fig. 2a shows the real part of the magnetic field profile (H_z) for an orthogonal electric dipole emitting at 560 nm and placed 10 nm above the surface of a flat Ag film. From the field profile, it is clear that the emitter couples to both LWs near the emitter and SPP modes that travel along the glass/Ag interface. If we replace the flat Ag mirror by a grooved metasurface with a periodicity of 150 nm, a groove depth of 100 nm and a filling fraction of 50%, we do not observe propagating SPPs along the surface. A detailed look at the field distribution in the near-field of the

emitter also shows that the LW fields are reduced in importance and this will be discussed further in the analysis of supplementary Fig. 4. Figure 2b also shows that the dipole above the HIM is emitting more efficiently to the far field. The same conclusions hold true for the emission of an electric dipole oriented parallel to the metal surface. The real part of the magnetic field profiles for these cases are shown in supplementary Fig. 2c and Fig.2d respectively.

From a practical perspective, it is irrelevant whether energy dissipates in the metal via LWs or SPPs as both generate undesired loss. It is thus useful to analyze the effect of a HIM on the far-field emission of a dipole by quantifying the fraction of the dipole power that is absorbed by the metal. To do so, we simulate the emission of an electric dipole emitting at wavelength of 560 nm placed in the organic host above a high-impedance metasurface consisting of silver patterned with a subwavelength array of grooves with a periodicity of 150 nm, a width of 75 nm and depth of 100 nm. We compare the results to those for a flat Ag mirror. We perform the simulations with a commercial Finite-Difference Time-Domain package (Lumerical FDTD Solutions). The distance between the dipole and mirror is changed from 10 to 160 nm. There are two power monitors below and above the dipole measuring the power absorbed in the metal (undesired) and the total radiated to the far field (desired) respectively. The simulation size is chosen to be relatively large (40 μm x 40 μm) to ensure a complete decoupling/dissipation of the SPP into the metal within the simulation volume. We define a quantity termed the loss ratio as the ratio of the power going through the monitor below the dipole to the total radiated power by the dipole. Supplementary Fig. 3a shows the loss ratio for an electric dipole oriented parallel to the surface of a smooth Ag surface as a function of dipole distance from the mirror surface. The loss ratio is substantially lower for the case of the high-impedance metasurface when the dipole distance is less than 60 nm. The peak in the loss ratio occurs at a distance of 80 nm for the metasurface due to the fact that the dipole is now placed close to the antinode of a standing wave profile created by the reflection of a normally-incident plane wave from the surface. Supplementary Fig. 3b shows the loss ratio as a function of the dipole distance for a dipole orientation perpendicular to the metal surface. For this orientation of dipole coupling of radiated photons to SPP modes is

more efficient. We can understand this intuitively by looking at the angular emission as shown in supplementary Fig. 5b, which is more directed along the surface. As a result, the loss ratio for this dipole orientation is above 80% for dipole distances smaller than 120 nm when placed above a smooth Ag film. If we replace the smooth Ag mirror by a high-impedance metasurface the loss ratio is dropped below 10% for all dipole distances in the range from 10 to 160 nm.

To understand how the HIM can improve the electrode performance, it is also worth understanding the role of the non-radiative non-plasmonic modes in the quenching of light emission from emitters. The non-radiative non-plasmonic modes, also known as Lossy Waves, are highly-localized, high-spatial-frequency modes that physically capture the excitation of an image-dipole of the emitter-dipole. Dissipation of electromagnetic energy in the metal can occur due to the coupling to such modes on both smooth metal surfaces and the patterned high-impedance metasurfaces.

To quantify the contributions of the coupling to SPPs and LWs to the total quenching of the molecules, we performed full-field simulations of an electric dipole in 4 different scenarios. We start with the well-studied problem of an emitter above a smooth metal film. Then we analyze the impact of digging a groove underneath the emitter, and finally we show the impact of the additional grooves to create a HIM.

Supplementary Fig. 4a shows a simulation of an electric dipole emitter radiating at 560 nm and placed 10 nm above a flat Ag mirror. The orientation of this dipole is along the x-direction, i.e. parallel to the metal surface. The simulation in figure panel (a) clearly shows the excitation of SPPs propagating away from the emitter along the metal surface. The inset shows a zoom-in of the region near the emitter and highlights the excitation of highly-localized LWs. From this simulation, we calculate a high loss ratio of 70%. We find that 60% of the loss in the metal is attributable to SPP excitation and the remaining 40% is associated with the excitation of LWs.

Second, we analyze the emission for the same dipole emitter, but now placed above a single, 75-nm-wide groove carved into the metal (Panel b). In this case, the excitation of LWs is highly suppressed as the metal is removed directly underneath the emitter. As a result, the excitation of SPPs will constitute the dominant mechanism by which energy is dissipated into the metal. With the removal of one loss channel, one might expect the fraction of optical energy deposited in the metal to reduce significantly. However, the simulation indicates that the loss ratio is virtually unchanged (from 70% to 68%). This is explained by an increase in the SPP loss that compensates for the losses to LWs. The physical mechanism for the enhanced SPP excitation lies in the effective coupling of the emitter dipole to gap SPPs that resonate in the (approximately quarter gap-SPP wavelength deep) groove, followed by an effective coupling of the gap SPPs to SPP on the metal surface. The effective excitation of the gap SPPs is visible in the inset to panel b.

Third, we analyzed the emission of a dipole in the center of the air-gap above a periodic array of nanoscale grooves (panel c). This geometry shows the important role of the periodic groove-arrays that have been added on either side of the central groove with the emitter. It can be seen that the SPPs on the surface are effectively decoupled and the loss ratio has now dropped to 26%. This shows the importance of creating a high-impedance metasurface(/properly designed groove-array) for decoupling the SPPs.

It is also important to look at emitters in other high-symmetry locations to achieve a more complete picture. Figure panel (d) shows a simulation of the emission the same emitter-dipole, but now placed just above the center of the metal beam of the metasurface. Here, the local environment to the emitter is hardly changed and the coupling to LWs is similar to that on a smooth metal film. The currently-designed, high-impedance metasurface does not make it possible to remove this loss component, although it may ultimately be possible to produce high-impedance metasurfaces with thinner teeth. However, the neighboring grooves again do help to decouple the SPPs from the surface and the loss ratio compared to the smooth surface is reduced to 34%. The results for emitters with different locations and orientations differ quantitatively, but are qualitatively the same;

Grooves underneath the emitter reduce the excitation of LWs and the groove-arrays significantly aid in the decoupling of SPPs. For example, the previously defined loss ratio for an electric dipole with the same orientation as what is shown in supplementary Fig. 4 that is placed just 5 nm away from surface of a flat mirror is 79%. Digging a single groove right below the dipole will reduce the loss to 69% while a periodic array of grooves would further reduce the loss ratio to 12%.

Supplementary Note 2: SPP transmission and reflection across a finite number of grooves

In this section we study the transmission of SPP waves across 10 deep subwavelength grooves with 150 nm periodicity and 50% filing factor. A SPP wave source at a wavelength of 560 nm is placed on the left side of the grooves. SPP waves travel along the silver/air interface and are decoupled to far-field radiation by means of grooves. Transmission and reflection coefficients of SPP waves are calculated as a function of grooves depth and shown in Supplementary Fig. 6a-b, respectively. The broadband nature of SPP decoupling by grooves can be seen from this figure. For example, at a groove depth of 80 nm the transmission is below 20% for wavelengths between 400 and 800 nm and the reflection amplitude is also less than 10%.

Supplementary Note 3: Electrical properties of HIM electrodes:

We can calculate the resistance of a periodic array of metallic nanobeams that make up the high-impedance metasurface. Such an array can be treated as a set of parallel resistors. In this calculation we consider the beam-array with periodicity P, a silver beam width of a, and height h. A small filling fraction of metal $f = a/P$ increases the resistance of a HIM that is given by $R_{HIM} = \rho\ (h/Af),$ where ρ is the resistivity of silver and A is the area of the HIM. Similarly, the resistance of a TCO layer of an equivalent thickness h can be written as $R_{TCO} = \rho\ (h/A)$. The magnitude of f for a practical HIM is between 0.1-0.9 while the resistivity of metals like Ag and Al is two or three orders of magnitude lower than a high-performance TCO. As a result, the electrical conductivity of a HIM will always be higher than those of a TCO spacer.

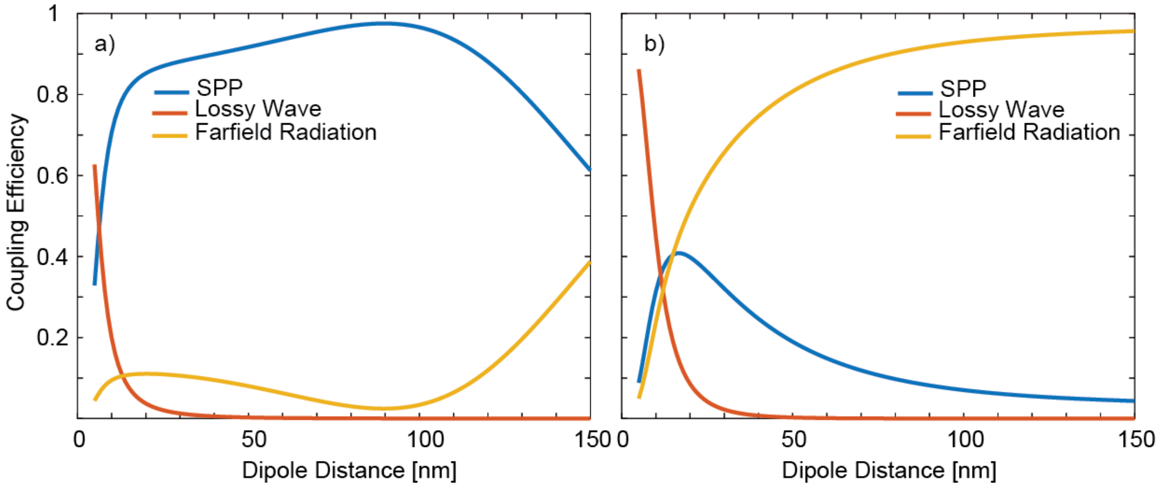

Supplementary Fig.1 The relevant decay channels for an electric dipole emitter above an Ag mirror. Dependency of the coupling efficiency for an electric dipole emitter into different decay channels. Here, we consider an emitter at 560 nm embedded in our organic host with a refractive index of 1.5. Panel a) shows the results for dipoles oriented parallel to the surface and b) perpendicular.

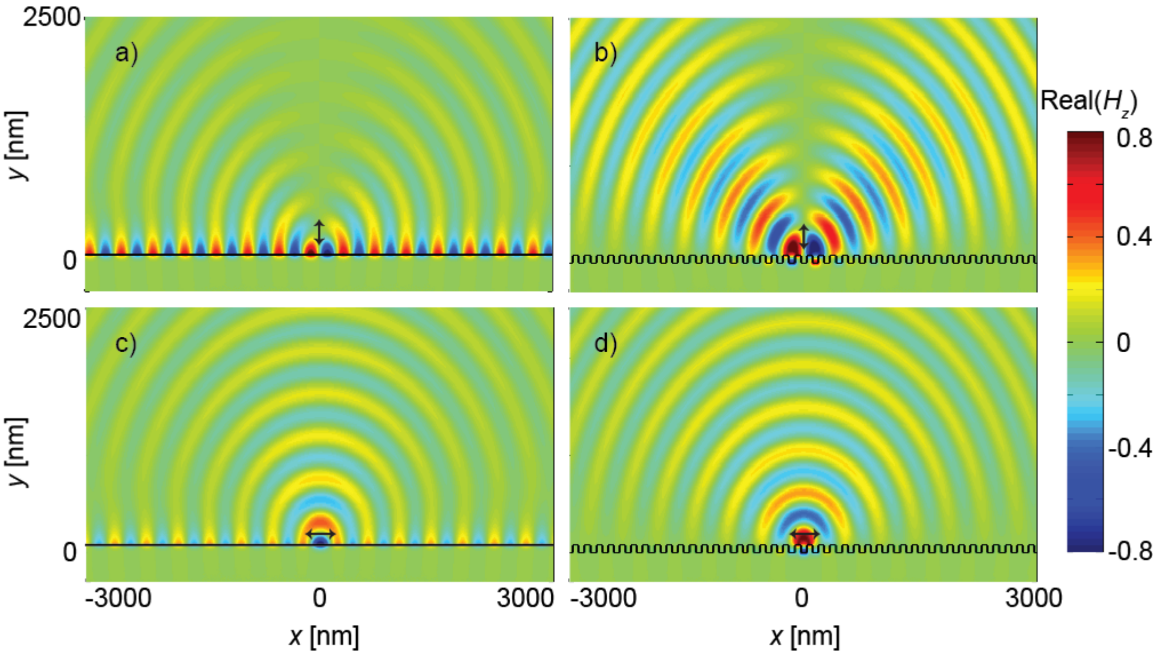

Supplementary Fig. 2. Optical simulation of electric dipole emission near smooth and patterned metal surfaces. a) Normalized real part of the magnetic field profile (H_z) of an electric dipole emitting at 560 nm and oriented perpendicular to the surface of a flat Ag mirror and b) a high-impedance metasurface. The same simulations, but now for c) a dipole oriented parallel to the surface of a flat Ag mirror and d) a high-impedance metasurface.

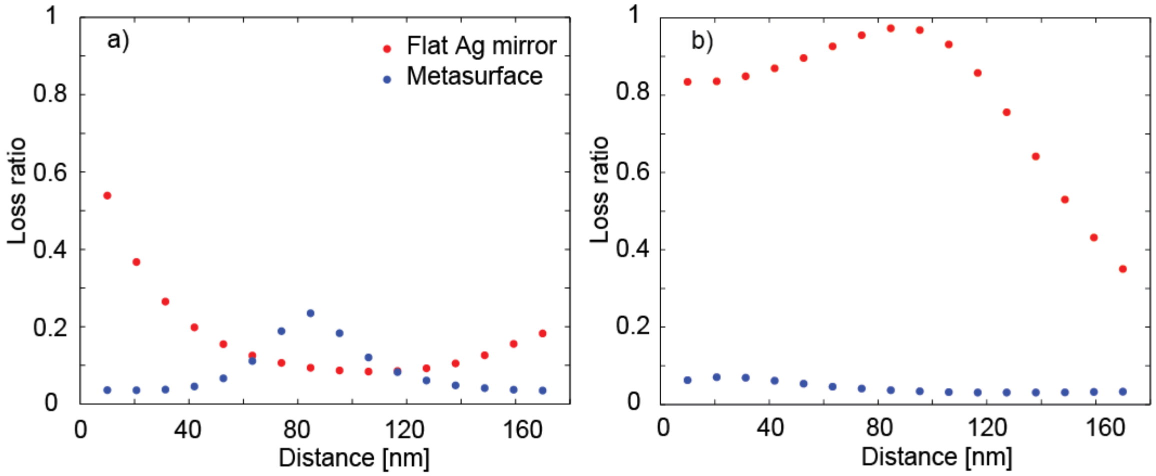

Supplementary Fig. 3. Height dependence of the loss ratio for electric dipoles above a flat Ag film and a high impedance metasurface. a) Calculation for an electric dipole emitter emitting at a wavelength of 560 nm and oriented parallel to the surface. b) Same calculation for a dipole oriented perpendicular to the surface. The high impedance metasurfaces in this example consists of grooves with depths of 100 nm and a filling fraction of 50%. The groove period taken as 150 nm.

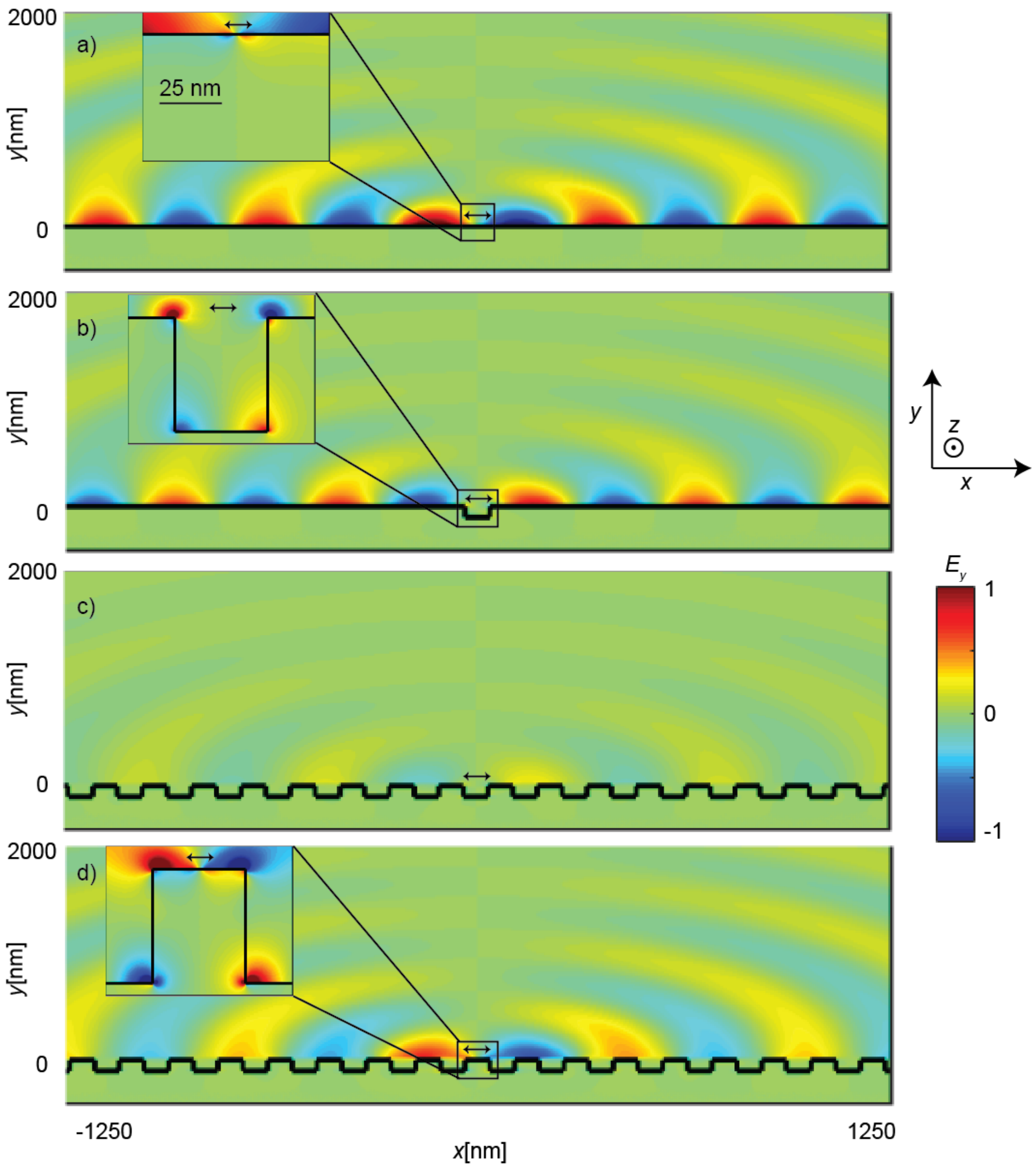

Supplementary Fig. 4. Impact of single and multiple grooves on the dissipation via SPPs and LWs a) Imaginary part of electric field profile Im(E_y) for an electric dipole emission above a flat silver mirror. The inset shows the zoomed in field profile near electric dipole showing the induced image dipole inside the Ag substrate. b) Electric field profile Im(E_y) for an electric dipole emission above a single groove. The inset shows the zoomed in field profile. c) Electric field profile Im(E_y) for an electric dipole emission above an array of grooves when places above the groove. d) Electric field profile Im(E_y) for an electric dipole emission above an array of grooves when places above the metal tooth.

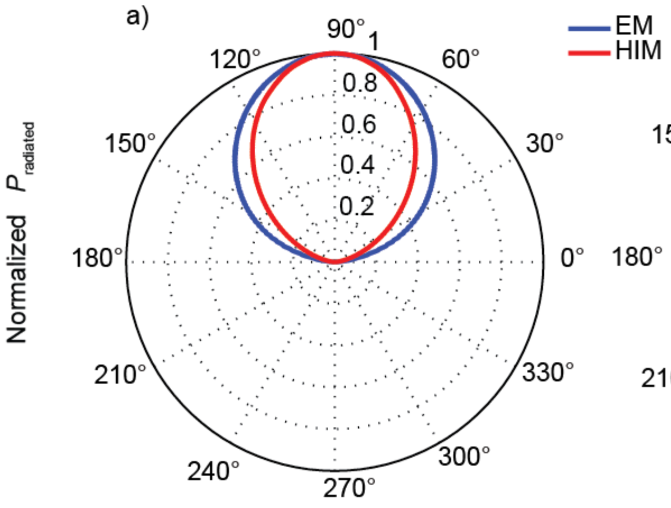

Supplementary Fig. 5. Far-field angular radiation pattern for an electric dipole above a smooth electric mirror (EM) and a high-impedance metasurface (HIM). a) Dipole oriented parallel to the surface of mirror. b) Dipole oriented perpendicular to the surface of the mirror. The dipole distance from the surface is 10 nm.

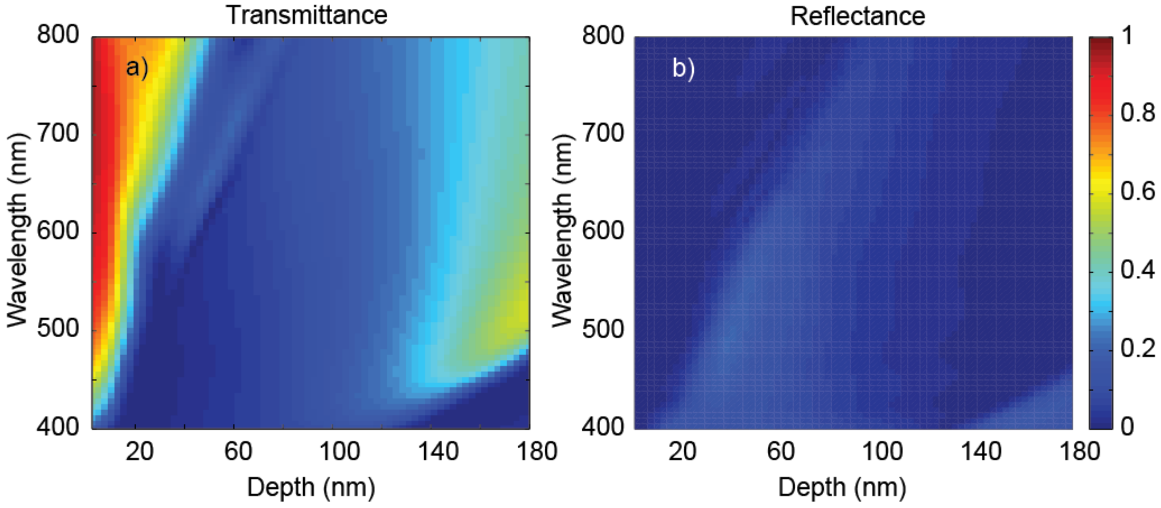

Supplementary Fig. 6. Optical simulation of the surface plasmon transmission and reflection across a finite number of subwavelength grooves. a) Maps of the calculated transmittance and b) reflectance of SPPs across a series of 10 grooves with the same dimensions as the grooves in Figure 2 of the main text (periodicity of 150 nm, and groove width of 75 nm) The vertical axis shows the free space wavelength and the horizontal axis provide the roove depth.

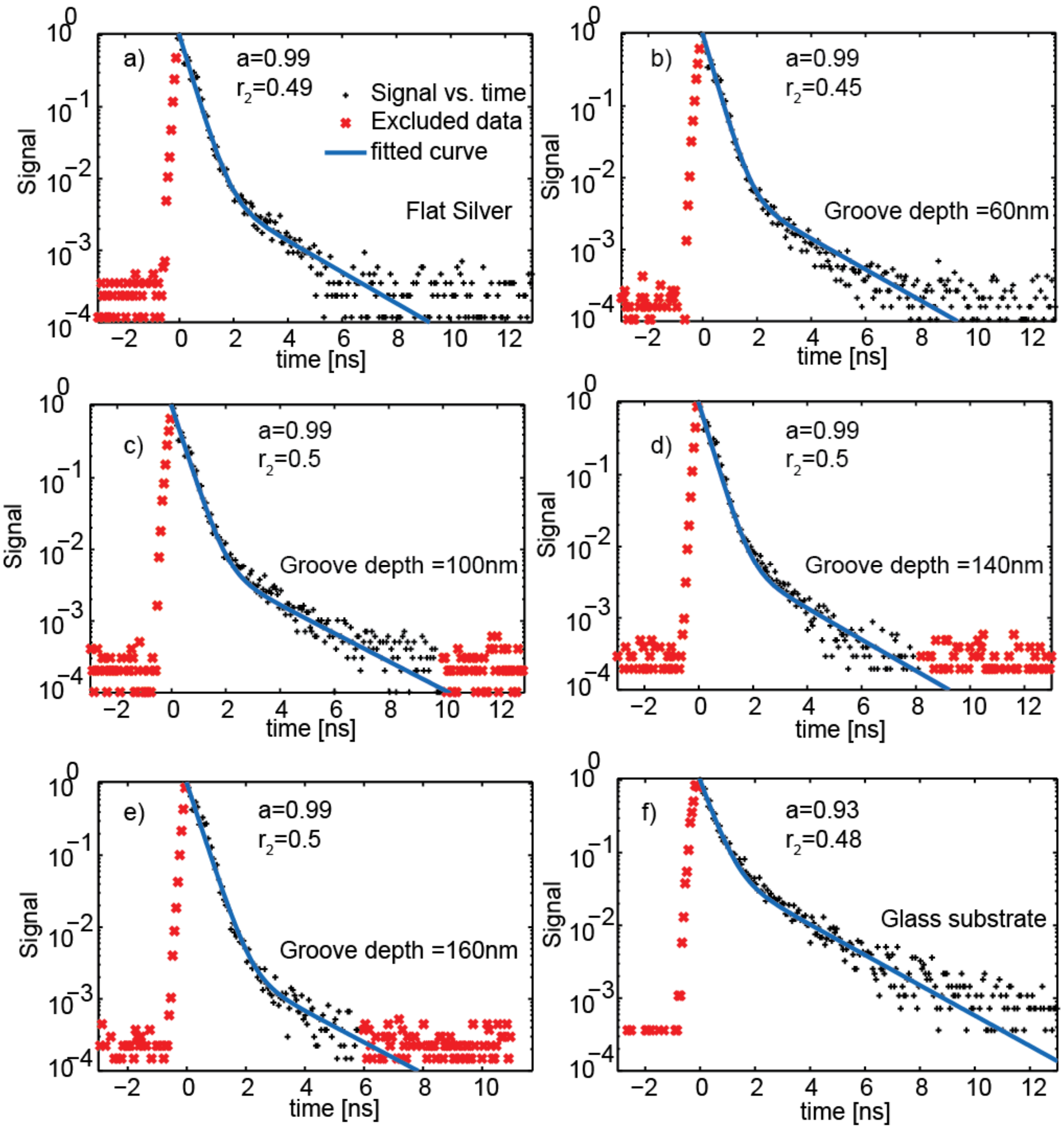

Supplementary Fig. 7. Time-resolved photoluminescence measurement of R6G dye molecules above different metallic surfaces. Time-resolved decay of 15-nm-thick R6G dye molecule with 5~10 nm spacer layer between dye layer and surface for **a)** flat silver surface **b)** metasurface with groove depths of 60 nm **c)** depth of 100 nm **d)** depth of 140 nm **e)** depth of 160 nm. **f)** Time-resolved measurement of R6G dye on a glass substrate. Each time trace is fitted with a double exponential function of the form $I(t) = ae^{-r_1 t} + (1-a)e^{-r_2 t}$, where I is the signal, r_1 and r_2 are the fast and slow rates, respectively, and a is the amplitude of the fast decay.

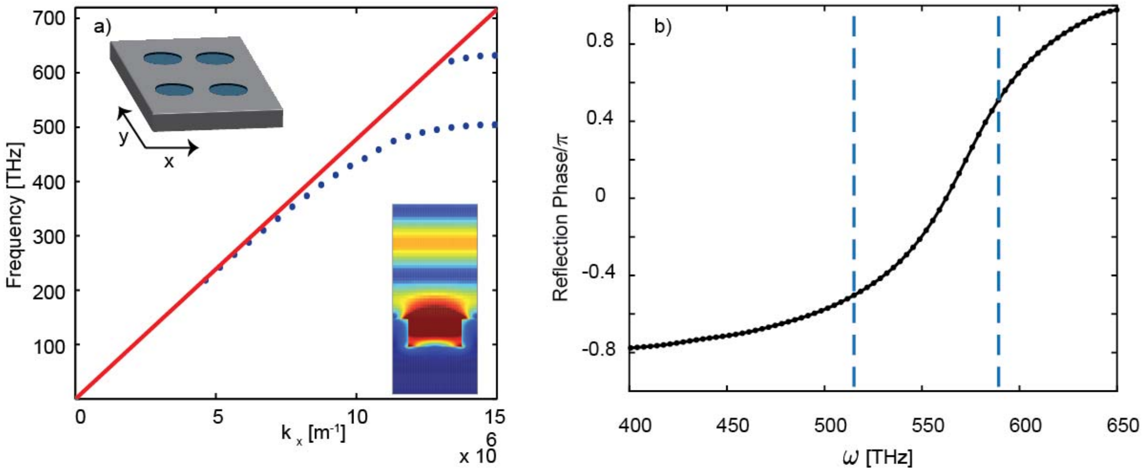

Supplementary Fig. 8. Band structure calculation for a periodic dimple array. a) Band structure of a periodic dimple array with periodicity of 210 nm, a SiO_2-filled dimple depth of 120 nm and a radius of 65 nm, along with the light line showing a band gap for a broad wavelength range from 485 to 600 nm. The inset shows the electric field profile magnitude for the case that normally-incident light at a wavelength of 560 nm is reflected back from the surface of a periodic dimple array. b) Simulated reflection phase for normally-incident light reflecting back from the same metasurface as in a) as a function of the frequency of the incident light wave.
